# Optimizing the production and affinity purification of HIV-1 envelope glycoprotein SOSIP trimers from transiently transfected CHO cells

**DOI:** 10.1371/journal.pone.0215106

**Published:** 2019-04-08

**Authors:** Albert Cupo, Victor M. Cruz Portillo, Paul Gelfand, Anila Yasmeen, P. J. Klasse, John P. Moore

**Affiliations:** Weill Cornell Medical College, New York, New York, United States of America; New York State Department of Health, UNITED STATES

## Abstract

We describe methods to improve the efficiency with which HIV-1 Envelope glycoprotein SOSIP trimer immunogens can be produced by transient transfection of ExpiCHO-S cells and then affinity purified using the trimer-specific human monoclonal antibody PGT145. The specificity of PGT145 for properly folded trimers allows for the facile, one-step, isolation of these immunogens in research laboratories. PGT145 columns are also valuable as a component of more complex purification processes in current Good Manufacturing Practice programs. However, we found that PGT145 purification was highly variable and markedly inefficient when used to process supernatants from transiently transfected ExpiCHO-S cells expressing the BG505 SOSIP.664 and other trimeric Env proteins. In contrast, no such problems arose when the same Env proteins derived from a stable CHO cell line were processed on the same PGT145 columns, or with transient transfection supernatants from 293F cells. An investigation of the ExpiCHO-S transfection system identified the presence of polyanions, including but perhaps not limited to dextran sulfate, in the Enhancer component of the transfection system. We hypothesized that these polyanions bound to the cationic PGT145 epitope on the trimers and impeded their ability to bind to the PGT145 affinity column. We found that replacing the Enhancer component with alternative culture medium supplements substantially increased the yield of PGT145-purifiable trimers, and we also confirmed that both dextran sulfate and the Enhancer component were indeed inhibitors of PGT145 binding to BG505 SOSIP.664 trimers in immunoassays. The presence of polyanions, including but not limited to nucleic acids, should be considered in other circumstances where PGT145 columns are less efficient than expected at purifying native-like trimers.

## Introduction

Native-like, recombinant soluble HIV-1 envelope glycoprotein (Env) SOSIP trimers are widely used as a vaccine development platform in programs aimed at inducing neutralizing antibodies (NAbs) [1, 2]. The prototype of this immunogen class, the BG505 SOSIP.664 trimer, has been produced under current Good Manufacturing Practice (cGMP) conditions for clinical trials and others are being made in this way for the same reason [3–5]. The most common method to produce SOSIP trimers in cGMP programs, and often also in the research laboratory setting, involves stably transfected Chinese hamster ovary (CHO) cells followed by affinity purification of the secreted trimers, initially via an anti-trimer monoclonal antibody (MAb)-based column [3, 6]. However, transient transfection of various cell types, including 293F and CHO, is commonly used in pre-clinical research, again followed by MAb affinity-column purification [1, 5, 7, 8]. One human MAb that is particularly useful for this purpose, PGT145, recognizes a trimer-specific (quaternary) epitope at the trimer apex [8–11]. PGT145 columns can therefore be used as a one-step method to purify native-like trimers from culture supernatants, at least in the pre-clinical environment [12–14].

The ExpiCHO-S transient transfection system offers advantages over 293F transfections, particularly as it allows the use of CHO cells for pre-clinical research that might advance to a cGMP program that is based on a stable CHO cell line [15]. We noted, however, that the recovery of various SOSIP trimers from PGT145 columns was highly variable when the trimers were produced in transiently transfected ExpiCHO-S cells. In contrast, no such problens were encountered when we similarly procesed supernatants from stable CHO cell lines or transiently transfected 293F cells. As both ExpiCHO-S cell transfections and PGT145 columns are otherwise highly useful methods, we sought to identify and overcome the sources of variability and thereby optimize the methodology. The outcome of our investigations was the identification and replacement of polyanions, particularly dextran sulfate, in a component of the ExpiCHO-S cell culture system that bind to the cationic PGT145 epitope on the trimers and compromise their association with the affinity column. Other polyanions, such as nucleic acids, might be similarly problematic under some conditions, as CpG oligo-deoxynucleotides (ODNs) are known to also occlude the PGT145 epitope on SOSIP trimers [16].

## Materials and methods

### Transient transfection of ExpiCHO-S or 293F cells

BG505 SOSIP.664 Env proteins were transiently expressed by co-transfecting separate plasmids that express *env* and *furin* genes [6, 7]. In some experiments, the proteins had a C-terminal hexa-Histidine tag (designated BG505 SOSIP.664-His) [13, 14]. The B41 SOSIP.v4.1-His construct was expressed in the same way [8, 12]. The transient transfection of FreeStyle 293-F cells (Thermo Fisher Scientific) for trimer production has been described previously [7, 8]. Trimers were transiently expressed in ExpiCHO-S cells using the ExpiFectamine CHO (Thermo Fisher Scientific) or FectoPRO (Polyplus-transfection SA) transfection reagents (see Results). ExpiCHO-S cells were cultured with ExpiCHO Expression Medium (Thermo Fisher Scientific) in a shaker incubator set at 135 rpm, 37°C and 8.0% CO_2_. The day before transfection, ExpiCHO-S cells were seeded at 3 x 10^6^ per ml in 500 ml of ExpiCHO Expression Medium. Following the manufacturer’s recommended protocol, 400 μg of Env plasmid, 100 μg of Furin plasmid and 1.6 ml of ExpiFectamine CHO reagent were mixed in 40 ml of cold Opti-PRO SFM (Thermo Fisher Scientific). Similarly, 320 μg of Env plasmid, 80 μg of Furin plasmid, and 800 μl of FectoPRO reagent were mixed in 50 ml of cold Opti-PRO SFM. A 250 μl quantity of FectoPRO booster was immediately added to the FectoPRO-transfected cultures. One day post-transfection, 120 ml of ExpiCHO Feed and 3.0 ml of ExpiCHO Enhancer were added to the ExpiFectamine-transfected cultures. Culture supernatants were harvested when cell viability dropped below 60% (day 6 to 14), clarified by centrifugation at 3,000 rpm for 30 min and filtered using a 0.2 μm filter (Thermo Fisher Scientific). The stable CHO cell line and its use to produce BG505 SOSIP.664 trimers have been described previously [6].

### Affinity purification of SOSIP trimers

Env proteins were purified by immunoaffinity chromatography using a 2G12 or PGT145 column [7, 8]. The 2G12 and PGT145 columns were made using CNBr-activated Sepharose 4B resin (GE Healthcare) coupled to 2G12 or PGT145 human MAbs (Polymun Sciences, Klosterneuburg, Austria). The culture supernatant was processed through the 2G12 or PGT145 affinity column at a flowrate of 1 ml per min for 24 h. When specified in Results, 2G12/SEC-purified trimers were processed through the PGT145 affinity column for 2 h while nutating at room temparture. The affinity column was washed with 2 column volumes of high-salt wash buffer (20 mM Tris-HCl, 500 mM NaCl [pH 8]), and Env proteins were eluted using 1 column volume of 3 M MgCl_2_ (pH 7.2). The eluted proteins were immediately buffer exchanged into 10 mM Tris-HCl and 75 mM NaCl (pH 8) using SnakeSkin dialysis tubing (molecular weight cutoff [MWCO] of 10 kDa) (Thermo Fisher Scientific) and further concentrated using a 100-kDa cutoff Vivaspin column (GE Healthcare). The 2G12 affinity-purified proteins were further purified by size-exclusion chromatography (SEC) on a Superdex 200 26/60 column (GE Healthcare) to isolate the trimer fractions and eliminate dimers and monomers. When PGT145 affinity columns were used without SEC, a modest amount of aggregated trimers was also sometimes observed, but their presence did not substantially affect the quantitative estimates of trimer recovery. When specified in Results, 100 ml of culture supernatant was buffer-exchanged into phosphate-buffered saline, pH 7.0 (PBS) using SnakeSkin dialysis tubing (10 kDa MWCO) prior to passage down the PGT145 column. Protein concentrations were determined using a bicinchoninic acid-based assay (BCA assay; Thermo Fisher Scientific). Env proteins purified by the 2G12/SEC or PGT145 methods were verified to be trimeric by blue-native polyacrylamide gel electrophoresis (BN-PAGE), as described previously [7, 8].

### Enzyme-linked immunosorbent assays (ELISA) for detecting PGT145-reactive trimers

The ELISA used to detect and quantify the production of PGT145-reactive SOSIP trimers has been described previously [3, 16]. Briefly, transfection culture supernatants were harvested and added to pre-blocked ELISA wells containing 200 ng of absorbed MAb 2G12, to allow the capture of all Env proteins. In an alternative ELISA, 100 ng of 2G12/SEC-purified trimers were added to pre-blocked ELISA wells containing 200 ng of absorbed MAb PGT145, to allow trimer capture. When specified in Results, the input trimers were mixed with dextran sulfate (Sigma-Aldrich, molecular mass 40 kDa) for 30 min at room temperature before addition to the wells for 1 h. In both assays, the unbound material was romoved by washing with PBS plus 0.01% Tween-20, before captured Env proteins were detected by the addition of biotin-labeled PGT145 (2G12 capture-ELISA) or biotin-labeled 2G12 (PGT145 capture ELISA), at 1 μg/ml followed by Poly-HRP streptavidin (Thermo Fisher Scientific). The color reactions were developed and stopped using the 1-Step Ultra TMB-ELISA substrate solution (Thermo Fisher Scientific) as per the manufacturer’s instructions, and the optical density was read at 450 nm. After background subtraction, a sigmoid function with variable slope was fitted to the 2G12 capture-ELISA data in GraphPad Prismv8.0. The BG505 SOSIP.664 trimer concentration in the transfection culture supernatants was extrapolated from a standard curve derived using known concentrations of 2G12/SEC-purified BG505 SOSIP.664 trimers diluted in supernatant from non-transfected ExpiCHO cells.

### Surface plasmon resonance (SPR) analysis of MAb binding to SOSIP trimers

SPR analysis of antibody binding to immobilized SOSIP trimers has been described previously [14, 17]. Briefly, affinity-purified C-terminally His-tagged trimers were captured by an anti-His Ab (GE Healthcare), which was coupled to CM5 chips, to trimer immobilization levels (*R*_*L*_ values) of 250 RU (response units). The 2G12 and PGT145 MAbs were injected at 500 nM with or without a prior injection of dextran sulfate (Sigma-Aldrich, molecular mass 40 kDa) at 1000 mg/L (25 μM) for 30 s. Association and dissociation were each monitored for 200 s at a flow rate of 50 μl/min. After each cycle, the anti-His surface was regenerated by injecting a single pulse of 10 mM glycine (pH 2.0) for 60 s [14].

### Error estimations

**Fig 1:**
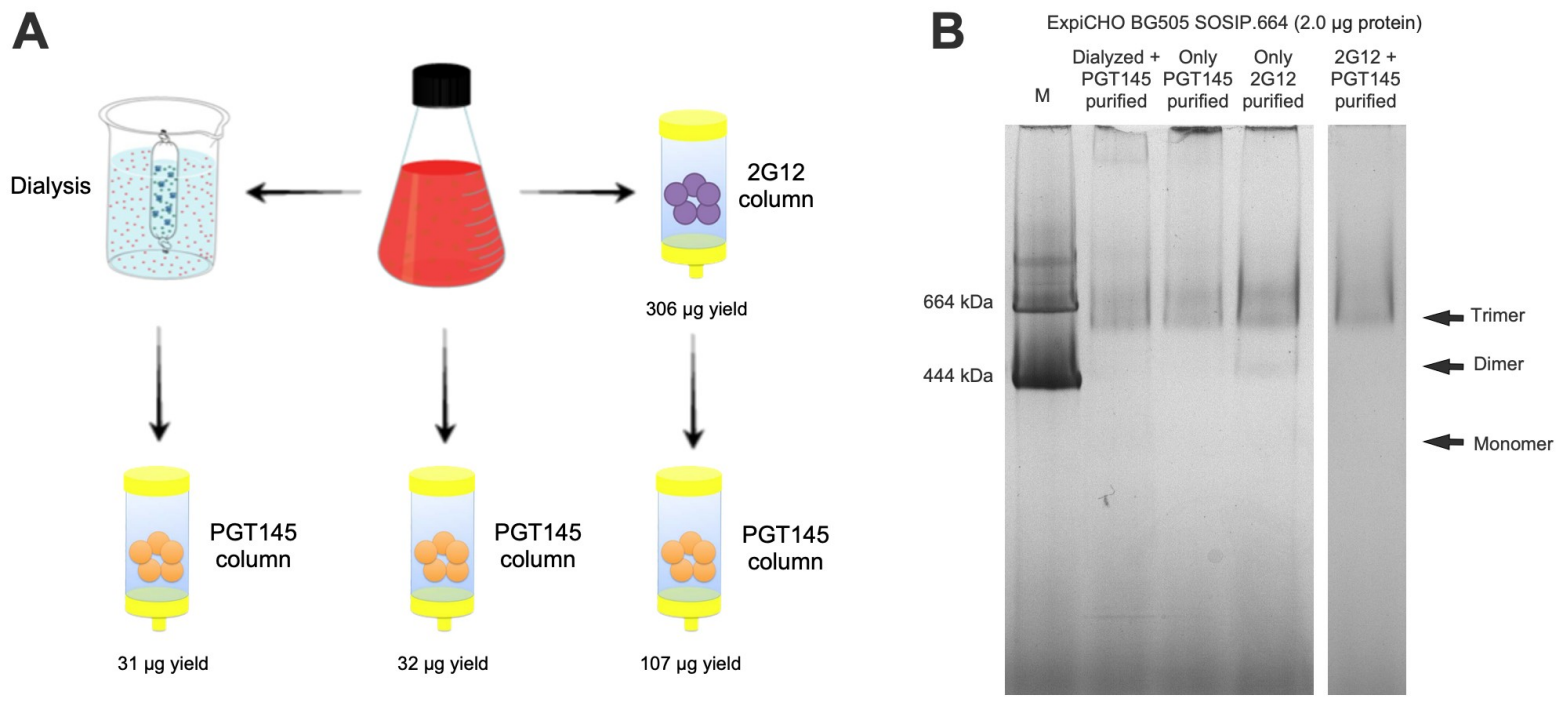
Purification scheme and analysis of BG505 SOSIP.664 trimers in ExpiCHO-S cultures. **(**A) The culture supernatant (red flask) was processed via the affinity columns, in the manners indicated by the arrows, leading to the purified trimer yields recorded below each column. (B) Coomassie blue-stained BN-PAGE analysis of ExpiCHO-S expressed BG505 SOSIP.664 trimer purified by the different affinity columns. The molecular masses of marker (M) proteins (thyroglobulin and ferritin) are indicated.

**Fig 2:**
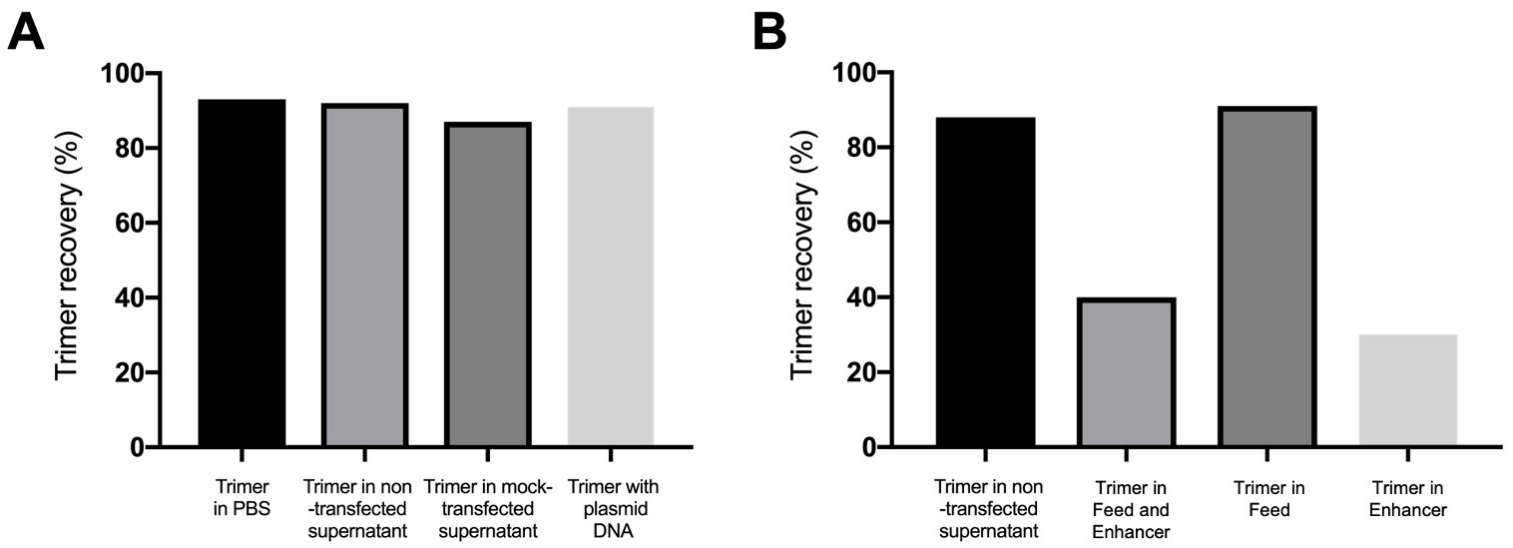
Effects of plasmid DNA and transfection components on PGT145 purification. Aliquots of 2G12/SEC-purified BG505 SOSIP.664 trimers were incubated as indicated and then passed down a PGT145 column, and the yields recorded as a percentage of the initial amount present (300 μg). (A) Incubation in PBS, or with an ExpiCHO-S culture supernatant from non-transfected or mock-transfected cells, or with 500 μg of the DNA expression plasmid as indicated. (B) Incubation in ExpiCHO-S cell culture supernatant, without or with the Feed and Enhancer supplements, as indicated.

**Fig 3:**
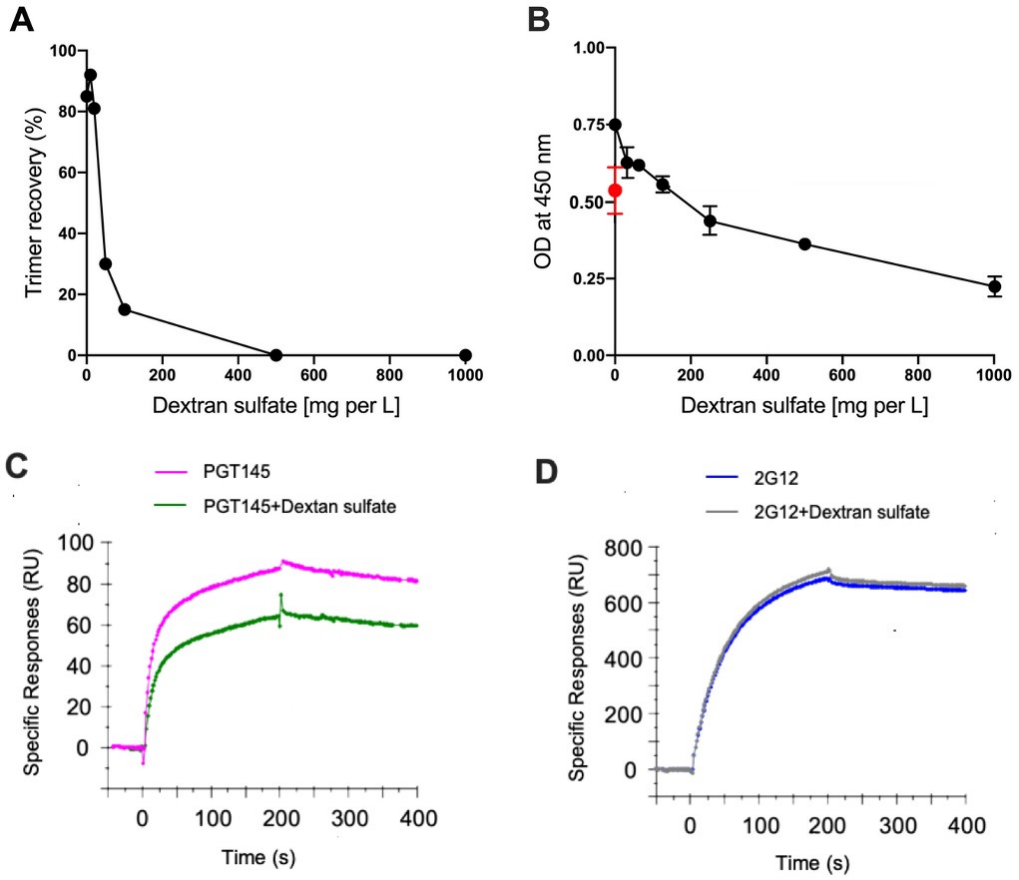
Effect of dextran sulfate on trimer purification via PGT145. (A) Aliquots of 2G12/SEC-purified BG505 SOSIP.664 trimers was incubated with dextran sulfate and then passed down a PGT145 column, with the yields recorded as a percentage of the initial amount (300 μg). (B) Aliquots of 2G12/SEC-purified BG505 SOSIP.664 trimers were incubated with the indicated dextran sulfate concentrations and then added to PGT145-coated ELISA wells before captured trimers were detected using biotinylated 2G12. The red dot indicates the optical density reading when trimers were instead incubated in the Enhancer component. The data points shown are the means ± s.e.m. of triplicate measurements. (C, D) SPR analysis of the effect of dextran sulfate on the subsequent binding of PGT145 or 2G12 to immobilized BG505 SOSIP.664 trimers. Dextran sulfate at 1000 mg/L (25 μM), or running buffer only, was injected for 30 s followed by 500 nM of either PGT145 or 2G12. The sensorgrams show median signals of bound antibody from three replicate cycles on the y-axis (RU) over time for 200 s each of association and dissociation. The signal in the control channel (i.e., with or without dextran sulfate) was subtracted from the corresponding curve. The values recorded in the text are means ± s.e.m. for triplicate determinations, in all cases.

**Fig 4:**
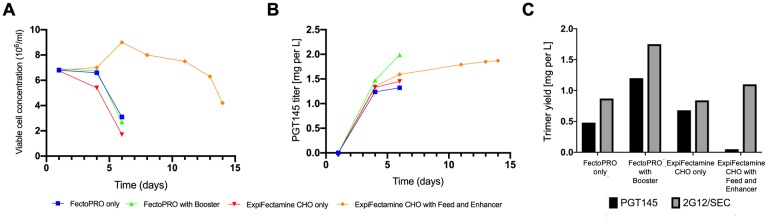
Production and purification of BG505 SOSIP.664-His trimers in ExpiCHO-S cultures. (A) The viable cell concentration was monitored using Trypan Blue exclusion over time in four ExpiCHO-S cultures containing the indicated transfection conditions. (B) The yields of PGT145-reactive trimers, measured by ELISA, in the same cultures. (C) The yields of 2G12/SEC- and PGT145-purifiable trimers from culture supernatants collected when the cell viability dropped below 60%.

The data shown in Figs 1–4 and Tables 1–2 are generally derived from single experiments but are representative of the outcomes of other experiments of a comparable design (e.g., the use of a different SOSIP trimer and/or a minor variation to the culture and/or purification conditions). Within each experiment, the endpoints determined by ELISA, including derivative values such as % trimer recovery, are based on triplicate wells that typically yield optical density values that vary from the mean by ± 0.72% (see, e.g., Fig 3B). These error estimates are reflected in the reported values. Protein concentrations determined using the BCA assay are typically subject to variations in the range 0.1–0.3%, which have a corresponding impact on the estimates of derivative values. Error estimates for SPR data are included in the legend to Fig 3C and 3D.

**Table 1:** BG505 SOSIP.664 trimer expression and purification in CHO cells.

Expression conditions	PGT145 yield (mg per L)	2G12/SEC yield (mg per L)
Stable expression using CHO-K1 cells with the Flp-In system	3.5–4.3	9.5–10
Transient expression using ExpiCHO-S cells with Expifectamine CHO with Feed and Enhancer	0–0.1	3.0–3.5

**Table 2:** Production and purification of SOSIP trimers under different conditions.

	BG505 SOSIP.664-His		B41 SOSIP.v4.1-His	
Transfection conditions	PGT145 yield (mg per L)	2G12/SEC yield (mg per L)	PGT145 yield (mg per L)	2G12/SEC yield (mg per L)
ExpiCHO-S with Expifectamine CHO with Feed and Enhancer	0.05	1.1	0.10	1.2
ExpiCHO-S with FectoPRO with Booster	1.2	1.75	1.7	2.1
293F with 293fectin	0.62	0.3	1.4	1.1

## Results

### Reduced trimer yield from ExpiCHO-S cell transfections

Our original method for purifying SOSIP trimers from the supernatants of transiently transfected 293T or 293F cells was based on the 2G12 anti-Env MAb followed by SEC [7]. The 2G12 MAb recognizes native-like trimers but also other forms of Env (e.g., monomers and dimers), which means that the trimer fraction of the column eluate must be separated by an SEC column step [7]. The PGT145 MAb is, however, completely trimer-specific, which offers the advantage, at least in laboratory research programs, of eliminating the need for an SEC column [10–14]. (In practice, we do usually process PGT145-purified trimers through an SEC column anyway, to remove a small amount of aggregates [8]). For a period of approximately 4 years, we used the PGT145 column method successfully, with or without SEC, to purify multiple SOSIP trimers from transiently transfected 293T or 293F cells, or from stable CHO cell lines [8, 12–14]. We did, however, occasionally encounter examples of unexpectedly low yields from PGT145 columns, without being able to identify why. Anecdotally, we noted that absorbing SOSIP trimers overnight onto PGT145-sepharose beads in suspension, rather than on a column, could increase trimer binding to, and then elution from, the purifying MAb. The implication was that the trimer-PGT145 association stage could be inefficient, at least under some conditions.

The problems with PGT145 affinity purification were particularly pronounced when we evaluated the ExpiCHO-S transfection method. The greater volume of cells and the more prolonged culture conditions allow SOSIP trimers to be produced and purified in multi-mg amounts from a single ExpiCHO-S transfection, whereas 293F transfections typically yield < 1 mg. The outcome is that an ExpiCHO-S transfection can provide enough SOSIP trimer for, e.g., an entire macaque immunization experiment, a useful feature. We soon found, however, that trimer yields from PGT145 affinity columns could be unacceptably low, compared to the 2G12/SEC method. An example is provided by an initial investigation of the problem (Fig 1A). Here, 100 ml aliquots of a supernatant from BG505 SOSIP.664-transfected ExpiCHO-S cells were passed through a PGT145 column, with or without prior dialysis (10 kDa MWCO), or instead passed through a 2G12 column. The Env protein yields from the PGT145 column were 32 μg and 31 μg, with and without dialysis, but 306 μg of Env was recovered from the 2G12 column. The 2G12 column eluate contains a considerable amount of non-trimeric Env proteins (Fig 1B), but when it was passed down the PGT145 column, 107 μg of trimer was recovered (Fig 1A). The ~3.5 fold increased recovery (107 *vs*. 31 μg) implies that the 2G12 column had removed an inhibitor that was preventing trimer association with the PGT145 column. The lack of benefit of the pre-dialysis stage indicates that the inhibitor’s mass was > 10 kDa.

The outcome of the ExpiCHO-S transfection experiments was inconsistent with our experience with CHO cell lines and 293F cell transfections. Thus, when the same BG505 SOSIP.664 Env protein was produced in the latter two systems, the amounts of trimers recovered by the 2G12/SEC and PGT145 column methods were comparable (Table 1).

### Identification of an inhibitor to PGT145 purification

Our initial hypothesis to explain the unusual problem associated with ExpiCHO-S transfection was that a nucleic acid such as DNA acted as an inhibitor of trimer binding to the PGT145 column. We were aware that CpG ODNs could bind to SOSIP trimers and occlude the PGT145 epitope [16]. We therefore reasoned that considerable amounts of DNA might be present in transfection supernants, either derived from the expression plasmid or released from dead or damaged cells. To test this idea, we purified BG505 SOSIP.664 trimers by the 2G12/SEC method, incubated 300 μg aliquots with or without 500 μg of the expression plasmid (i.e., DNA) or with the supernatant media from a non-transfected or a mock-transfected (Furin but no Env plasmid) ExpiCHO-S cell culture, and then processed the trimers on the PGT145 column. In each case, 87–93% of the trimer was recovered (Fig 2A). Thus, neither the expression plasmid DNA nor any component of a standard ExpiCHO-S culture supernatant inhibited trimer recovery on the PGT145 column.

The ExpiCHO-S transfection method involves the addition of Enhancer and Feed components to the CHO cells to maintain the viability of the cells. Following the manufacturer’s instructions, we next assessed whether these components affected PGT145 recovery. While the unsupplemented transfection supernatant again had no substantial effect, the addition of the Enhancer plus Feed components or only the Enhancer component reduced trimer recovery to 40% and 33% of the input, respectively (Fig 2B).

A review of the patent literature revealed that the ExpiCHO-S Enhancer component contains, *inter alia*, an unspecified amount of dextran sulfate, a polycation that prevents cell aggregation into clumps during the multi-day span of the post-transfection culture [18]. Dextran sulfate with a mass of 40 kDa has been found to be optimal for improving CHO cell growth when producing MAbs [19]. Dextran sulfate is known to inhibit the binding of monoclonal antibodies to the third variable (V3) region of recombinant and cell surface HIV-1 gp120 Env proteins [20]. We therefore investigated whether the inhibitory effect of the Enhancer component could be mimicked by titrating pure dextran sulfate (40 kDa, Sigma-Aldrich) to 2G12/SEC-purified BG505 SOSIP.664 trimers, and found that dextran sulfate concentrations > 20 μg/ml inhibited trimer purification via the PGT145 column in a dose-dependent manner (Fig 3A). We also showed that the same dextran sulfate stock and the Enhancer component each inhibited PGT145 binding to 2G12/SEC-purified BG505 SOSIP.664 trimers in an ELISA (Fig 3B). An SPR analysis confirmed that dextran sulfate inhibited the binding of PGT145, but not 2G12, to BG505 SOSIP.664 trimers immobilized to the chip via their C-terminal His-tags (Fig 3C). At the end of the association phase after dextran sulfate pre-injection, PGT145 binding was reduced to 70 ± 0.9% of the control level (no dextran sulfate) whereas 2G12 binding was unaffected at 100 ± 2%. Dextran sulfate injection for 30 s was sufficient for maximal binding to the trimer (25 ± 3.0 RU corresponding to 0.9 ± 0.1 molecule per trimer; not shown). Note that the competitive conditions that apply to an SPR analysis do not fully mimic what happens in the affinity purification setting. Nonetheless, the ELISA and SPR data show that dextran sulfate, which is present in the Enhancer component, is an inhibitor of PGT145 binding to the the trimers. It is possible that other, unidentified constituents of the Enhancer may also be inhibitory. Taken together, we propose that the Enhancer component of the ExpiCHO-S medium, particularly its dextran sulfate content, accounts for the observed impediment to efficient PGT145 affinity purification.

### Optimizing the ExpiCHO-S transfection method for PGT145 recovery

To improve the production of SOSIP trimers in transfected ExpiCHO-S cells, we expressed a BG505 SOSIP.664-His construct in these cells using four different transfection conditions: ExpiFectamine CHO, ExpiFectamine CHO with Feed and Enhancer, FectoPRO and FectoPRO with Booster. Using the ExpiFectamine CHO reagent and with its supplements for ExpiCHO-S cells is recommended by the manufacturer (Thermo Fisher Scientific); the FectoPRO reagents were purchased from a different supplier, Polyplus-transfection SA. Then the four ExpiCHO-S cultures were monitored over time. In the ExpiFectamine cultures, cell viability crashed/cells died after day-4 unless Feed and Enhancer were included, in which case the culture remained viable until day-14 (Fig 4A). However, when SOSIP trimer production was measured over time using a PGT145-based ELISA, we made two relevant observations. First, there was only a small increase (17.6%) in trimer production in the ExpiFectamine CHO with Feed and Enhancer culture between days 6 and 14; second, that the greatest amount of PGT145-reactive trimer detected by the ELISA on day-6 was in the FectoPRO with Booster culture (Fig 4B), and essentially no trimers could be purified from the ExpiFectamine CHO with Feed and Enhancer culture (Fig 4C). Comparing the ELISA data for PGT145-reactive trimers (Fig 4B) and the yields of PGT145-purifiable trimers (Fig 4C) clearly shows that the problem associated with the latter culture method is that, while the trimers are present in the culture supernatants, they cannot be purified via the PGT145 column.

We next expressed the BG505 SOSIP.664-His trimer in ExpiCHO-S cells for 14 days, using either the ExpiFectamine CHO with Feed and Enhancer or the FectoPRO with Booster systems, and then purified the trimers by the 2G12/SEC or PGT145 column methods (Table 2). For comparison, we also transfected 293F cells with the same plasmid, using the 293fectin system. The highest trimer yields were from ExpiCHO-S cells cultured with FectoPRO with Booster, and although more trimers were recovered using the 2G12/SEC column method, the PGT145 affinity column also worked efficiently under these conditions (Table 2). The switch to the FectoPRO with Booster method increased the trimer yield from the PGT145 column 24-fold (0.05 to 1.2 mg/L). Neither the problem nor the solution to the inefficient PGT145 purification of trimers from ExpiCHO-S transfection supernatants was unique to BG505-based SOSIP trimers. Thus, when we attempted to purify B41 SOSIP.v4.1-His trimers expressed by ExpiFectamine transfection of a CHO culture with Feed and Enhancer, the recovery from PGT145 columns was only ~8% of that obtained via the 2G12/SEC method, but when the ExpiCHO-S cells were transfected using FectoPRO with Booster, the yield from the PGT145 column was increased 17-fold (from 0.1 to 1.7 mg/L) compared with the ExpiFectamine transfection (Table 2).

## Discussion

Here, we show that the otherwise considerable benefits of the ExpiCHO-S cell transient transfection method for producing multi-milligram amounts of PGT145-purifiable SOSIP trimers are compromised by the presence of an inhibitor in the Enhancer component of the manufacturer-recommended culture system. This inhibitor impedes the binding of SOSIP trimers to the PGT145 affinity column. The most likely culprit is the polyanionic anti-clumping agent dextran sulfate, which is present in the Enhancer component and which we showed does act as an inhibitor of PGT145 binding to SOSIP trimers in immunoassays. The mechanism is most probably a charge-based interaction between the polyanion and the cationic apex of the trimer where the PGT145 epitope is located. A similar PGT145 epitope-occluding effect was previously seen with CpG ODNs, a DNA-based polyanionic adjuvant [16].

We consider it possible that DNA, or perhaps RNA, released from dead or moribund cells could also be problematic for PGT145 affinity columns under some conditions, although we did not observe an inhibitory effect in a spiking experiment when purified DNA (an expression plasmid) or a mock transfection culture supernatant was mixed with SOSIP trimers. The impact of nucleic acids should be explored if PGT145 affinity columns work poorly under circumstances when a high level of cell death is seen, for example as a result of electroporation-based transfection [21]. The presence of polyanions in general should also be considered, as it is possible that compounds such as, but not limited to, dextran sulfate are components of various complex media used in GMP programs. The implications of the presence and properties of highly charged media components such as, but not limited to, dextran sulfate could extend beyond the narrow confines of how to purify HIV-1 Env SOSIP trimers to other protein products, including monoclonal antibodies. We noted, for example, that adding a high concentration (500 mg/L) of dextran sulfate to a PGT145 affinity column seriously compromised the future performance of that column, presumably via charge-based interactions.

The solution we identified was to eliminate the need for the ExpiCHO-S cell Enhancer component by using instead a different transfection medium culture system, FectoPRO with Booster, with the ExpiCHO-S cells. Other such systems could probably also be identified, but we did not conduct an exhaustive survey of available commercial products. We also note that the FectoPRO with Booster system is ~40% cheaper than its ExpiFectamine CHO with Feed and Enhancer counterpart. This methodology change would therefore also provide cost savings when producing multi-millgram amounts of SOSIP trimers by transient transfection on a research laboratory scale, under circumstances when the creation of a stable cell line is not justified.
